# Editorial: Application of next-generation sequencing in clinical settings

**DOI:** 10.3389/fgene.2025.1700975

**Published:** 2025-10-09

**Authors:** Zhi Chai, Han Mei, Quan Li, Iram Maqsood, Jingxian Cai, Marilyn M. Li, Feng Xu, Xiaonan Zhao

**Affiliations:** ^1^ Icahn School of Medicine at Mount Sinai, New York, NY, United States; ^2^ Asimov Inc., Boston, MA, United States; ^3^ University Health Network, Toronto, ON, Canada; ^4^ University of Maryland, College Park, MD, United States; ^5^ Regeneron Pharmaceuticals Inc., Basking Ridge, NJ, United States; ^6^ The Children’s Hospital of Philadelphia, Philadelphia, PA, United States; ^7^ Baylor College of Medicine, Houston, TX, United States

**Keywords:** next-generation sequencing - NGS, clinical genetics, precision medicine and genomics, rare disease, genomic

## Introduction

Next-generation sequencing (NGS), or high-throughput sequencing, has transformed the landscape of clinical genetics by enabling rapid, scalable, and cost-effective analysis of DNA and RNA. NGS facilitates the detection of low-frequency and complex variants across a broad spectrum of diseases, from rare monogenic disorders to complex polygenic traits (Satam et al., 2023). With the growing application of NGS-based diagnostics, regulatory frameworks such as those from the FDA and Centers for Medicare and Medicaid Services (CMS) have expanded to support clinical-grade tests and companion therapeutics (Karlovich and Williams, 2019). These advances have made NGS an imperative tool in precision medicine, influencing diagnostics, prognosis, and treatment across medical specialties (Arikat and Saboor, 2024).

This Research Topic brings together nine original contributions highlighting the diverse and expanding clinical applications of NGS, from technology development and bioinformatic innovation to disease diagnosis and case-based precision treatment.

## NGS technology development and diagnostic pipeline innovation

Sen et al. validated a clinically deployable long-read sequencing pipeline using Oxford Nanopore Technology. Their integrated approach managed to call variants in different types, namely SNP, structural variants, as well as those in homologous regions, with >99% concordance in benchmarking studies. Importantly, the pipeline resolved diagnostic dilemmas that short-read NGS could not, reinforcing its utility as a unified diagnostic tool for patients with suspected genetic disorders.

Corey et al. introduced a method to calculate maternal polygenic risk scores (PRS) using low-coverage whole-genome sequencing of cell-free DNA (cfDNA) collected during prenatal screening. PRS derived from cfDNA showed high concordance with matched genomic DNA (r ≈ 0.9), demonstrating that this non-invasive approach could enhance prenatal risk assessment without altering standard screening workflows.

Gorman et al. reported on a cohort of over 1,500 individuals tested with dual nuclear-mitochondrial genome NGS panels for suspected mitochondrial disease. Their 14.6% diagnostic yield, split nearly equally between nuclear and mtDNA variants, supports dual-genome panels as a first-tier approach. The study emphasizes the need for updated gene panels and consideration of tissue-specific testing to capture the full spectrum of pathogenic variants.

## Rare disease genomics and its impact on precision diagnosis

Zabihi et al. applied whole-exome and targeted sequencing to identify novel and known pathogenic variants in mucopolysaccharidosis (OMIM #309900, #252940, #253000, #252900, #252920) among consanguineous Iranian families. Six previously unreported variants were found across multiple lysosomal genes. Their study underscores the role of NGS in diagnosing recessive disorders and facilitating family-based screening and prevention strategies in high-risk populations.

Mansour-Hendili et al. described four patients from unrelated Maghreb families with SMVT deficiency (OMIM #618973), all carrying the same intronic variant in *SLC5A6*. Transcript-level validation reclassified this variant as pathogenic, opening avenues for treatment and genetic counseling. The recurrence suggests a possible founder mutation, emphasizing the importance of regional variant tracking.

Vecchio et al. expanded the mutational spectrum of IHPRF1 syndrome (OMIM #615419) by identifying deleterious biallelic variants in *NALCN*. Through *in silico* modeling, they demonstrated structural disruption of the encoded ion channel. This work illustrates how computational tools are complementary to NGS approaches in refining genotype-phenotype correlations for rare disorders.

Tamayo-Trujillo et al. reported a case of congenital Long QT Syndrome type 2 (OMIM #613688) in an Ecuadorian adolescent. NGS identified a known pathogenic variant in *KCNH2*, guiding the clinical decision to implant a cardioverter-defibrillator. Their case highlights how genomics can personalize risk stratification and therapeutic interventions in inherited cardiac conditions.

## Somatic mosaicism and complex variant interpretation

Tooming et al. presented three cases of PIK3CA-related overgrowth syndromes (OMIM #171834), characterized by post-zygotic somatic mosaic variants. Standard testing failed to detect pathogenic mutations, underscoring the need for tissue-specific or ultra-sensitive NGS approaches. Their study advocates comprehensive genomic profiling to improve diagnosis and management of mosaic disorders.

Yang et al. described a rare case of over 100 bilateral pulmonary ground-glass opacities, diagnosed as both primary tumors and intrapulmonary metastases. Using an 808-gene NGS panel and circulating tumor DNA (ctDNA) profiling, they uncovered tumor clonality and guided targeted treatment with osimertinib. This case illustrates the utility of NGS in both diagnosis and real-time therapeutic monitoring.

Together, this Research Topic of research articles reflects a wide wingspan of NGS applications, ranging from DNA, cfDNA, to mtDNA and transcriptome, from SNV to CNV, and from germline variants to somatic variants, demonstrating the growing impact of NGS across biomedical research and clinical practice. Leveraging a diverse range of NGS approaches–including long-read sequencing, low-pass WGS, targeted panels, and liquid biopsy, the studies demonstrate the versatility of NGS in addressing diverse clinical and scientific questions. From validating scalable pipelines to solving rare diagnostic puzzles and guiding individualized treatment strategies, the studies underscore NGS continues to evolve as an essential pillar of modern clinical practice. The inclusion of clinically relevant case reports further illustrates its practical value in real-world healthcare settings.
